# Supporting policymakers with a decision aid to evaluate the impact of e-cigarette flavour restrictions

**DOI:** 10.1186/s12961-026-01494-1

**Published:** 2026-06-15

**Authors:** Angela S. Attwood, Jennifer Ferrar, Mark J. Gibson, Martin Dockrell, Marcus R. Munafò, Jasmine N. Khouja

**Affiliations:** 1 School of Psychology and Neuroscience, University of Bristol, Bristol, United Kingdom; 2 Vice Chancellor’s Office, University of Bath, Bath, United Kingdom; 3 MRC Integrative Epidemiology Unit at the University of Bristol, Bristol, United Kingdom; 4 Department of Psychology, University of Bath, Bath, United Kingdom; 5 Improving Performance in Practice, iPiP, Warwick, United Kingdom

**Keywords:** Policy communication, E-cigarettes, Vaping, UK legislation, Flavours

## Abstract

**Background:**

The UK Government has introduced a range of measures to tackle the rise in youth vaping, including the power to regulate flavours. Evidence is needed to inform how these measures should be implemented, but communication between health researchers and policymakers can be difficult. Policymakers need a simple, easy-to-digest synthesis of evidence to make policy decisions that benefit the public.

**Methods:**

We designed a policy decision aid to support policymakers (commissioned by Public Health England), whereby existing data can be inputted to create a report (the decision aid). The report estimates whether restricting flavours other than tobacco or menthol flavours in e-cigarettes would have a net benefit due to reductions in youth vaping and smoking or a net detriment due to the negative impact on smoking cessation and relapse rates.

**Results:**

Using the available evidence on 13 November 2024, the model estimated that 125,034 non-smoking youth experiment with e-cigarettes as a result of flavoured e-liquid availability, and 841,302 smokers and ex-smokers do not smoke due to flavoured e-liquid availability. The model estimated that 48,764 non-smoking youth subsequently smoke as a result of flavoured e-liquid availability. The algorithm indicated that if only unflavoured, tobacco flavoured or menthol flavoured vapes remained on the market, there would be a detrimental impact on smoking rates in adults that would outweigh the number of young people protected from vaping and later smoking.

**Conclusions:**

This output suggests that restricting flavoured e-liquids in the United Kingdom could have a detrimental impact on public health when considering both youth vaping and smoking uptake due to flavoured vape availability. To provide timely advice to policymakers, the algorithm used is intentionally simple. The decision aid should be used alongside existing relevant evidence (e.g. from countries with similar regulatory environments) to inform policy decisions and/or used to highlight areas for research that warrant support. The reports have been used in policy documents and discussions, demonstrating an appetite for this type of communication aid. The tool can be used to assess this policy question within subpopulations and in other localities, and the framework can be adopted to address other policy questions.

**Supplementary Information:**

The online version contains supplementary material available at 10.1186/s12961-026-01494-1.

## Background

There have been substantial changes to e-cigarette regulation in the United Kingdom (UK) recently; in 2024, the UK government proposed a new Vaping Products Duty, a ban on disposable vapes (also known as e-cigarettes) and the Tobacco and Vapes Bill [1]. These policies were proposed in response to a rapid increase in youth vaping [2] and concerns that e-cigarettes may cause a range of health outcomes [3] and/or act as a gateway to smoking [4, 5]. The UK government received substantial advice on these policies from researchers in response to calls for evidence and consultations, and via written and oral evidence submitted to the Tobacco and Vapes Bill Committee. However, researchers often struggle to translate complex evidence into clear narratives, leaving policymakers to navigate the challenge of weighing both the potential benefits and possible harms of a new policy [6, 7].

Policymakers need to make countless decisions that may impact the health of the population they serve without necessarily being an expert on the health topic or the corresponding evidence base. In contrast, public health researchers are experts in their topic area and can have substantial knowledge that could be used to inform policy decisions, but it is often difficult to communicate this information effectively [6]. Key barriers to the use of evidence in policy decisions include evidence not being submitted at the right time to influence policy, a lack of clarity in the submitted evidence and policymakers not having the research skills needed to understand the evidence [8]. Furthermore, evidence needs to be integrated into context-sensitive decision-making processes to drive change [9].

In the context of the Tobacco and Vapes Bill, one proposal that has received particular attention is the introduction of new powers allowing Ministers to regulate the flavours of vapes. Although many in the UK Parliament have called for a complete flavour ban, those supporting this approach may not fully appreciate the potential unintended consequences highlighted in the research, especially given that policymakers are often not in a position to weigh the evidence themselves. Only 3 of the 56 pieces of written submissions sent to the Tobacco and Vapes Bill Committee included evidence from public health researchers on the impact of banning flavours.

Some submissions highlighted qualitative evidence from young people and adults; the response from the University of Stirling indicated that flavours contribute to the appeal of vaping for young people but noted evidence also shows flavours can help adults transition away from tobacco. Submitted evidence also stated that interviews with UK adults who smoked and vaped suggest that whilst some people may stop vaping, others may not stop smoking using e-cigarettes or may relapse if flavours (aside from menthol and tobacco) are banned [10]. This is particularly concerning given e-cigarettes are a popular and effective aid to stop smoking in England; 40% of respondents in the Smoking Toolkit Study claimed to have used an e-cigarette in their most recent quit attempt, and those using an e-cigarette (versus not) are nearly twice as likely to stop smoking [11].

Written evidence also referred to international findings that showed the utility of flavoured vapes in smoking cessation and potential consequences of a ban [12–15]. Evidence from other countries suggests that banning flavours (or allowing only tobacco and menthol flavours) could be effective in reducing the appeal of vaping to young people [16, 17], but there have been several studies that suggest this policy could also increase tobacco use and illicit product use [18–21].

Although these studies highlight the potential consequences of a flavour ban in the UK, policymakers may struggle to consolidate the findings, as they are not always equipped to compare the opposing impacts reported in complex evidence, particularly when studies use disparate designs. In addition, policymakers cannot solely rely on international evidence to make decisions, as the impact may not be the same due to the different local social and regulatory environments.

In 2020, we were commissioned by Public Health England (now the Office of Health Improvements and Disparities) to create a decision-making aid to be used by policymakers in conjunction with other evidence. The aid aimed to estimate the impact of a flavour ban on never-smoking youth versus adults who vape and smoke, whilst providing information in a simple, easy-to-digest report. We created a tool that would automatically produce this report (the policy decision aid) when new data from a range of sources is entered into the tool, thus enabling us to quickly provide policymakers with updated reports on the basis of the latest available data when needed. This tool provides brief, digestible guidance to policymakers who are developing e-cigarette policies in the UK, but the framework can be applied to subpopulations, other localities and other policy questions with context- and population-specific amendments.

## Methods

### Model structure

The policy decision aid was designed by the authors and refined in consultation with relevant stakeholders, including other UK vaping researchers, leads for national surveys that collect data on vaping, and UK policymakers interested in vaping regulations. The tool we created automatically produces an updated report when new data are entered. It applies a basic algorithm that uses the following inputs:

A. The extent to which flavours draw non-smoking youth to vape.

Let:

- a = The number of youth at smoking age in the UK
- b = The proportion of youth at smoking age who do not smoke
- c = The proportion of youth at smoking age who have never smoked but have vaped
- d = The proportion of the above who state they vape because of flavours
  $$A=a\times b\times c\times d$$

B. How many non-smokers who vape as a result of e-liquid flavour availability may then go on to smoke.

Let:

- a = The value of A
- b = The proportion of non-smoking youth who try vaping and later try smoking
  B=a×b

C. To what extent e-liquid flavour availability draws in smokers who would quit smoking using e-cigarettes (per year).

Let:

- a = The number of adult smokers in the UK
- b = The proportion of smokers who quit smoking because of e-cigarettes
- c = The proportion of smokers who vape who state that they would not quit or would smoke more if flavours were not available
  $$C=a\times b\times c$$

D. The number of e-cigarette users who state they would relapse to smoking if flavoured e-liquid were not available on the UK market.

Let:

- a = The number of ex-smokers in the UK
- b = The proportion of ex-smokers who vape
- c = The proportion of ex-smokers who state they would relapse to smoking if flavours were not available
  $$D=a\times b\times c$$

To estimate whether the number of non-smoking e-cigarette users introduced into the UK population because of flavoured e-liquid availability could outweigh the number of smokers and ex-smokers who might vape instead of smoke because of flavoured e-liquid availability, the following calculation is made:

$$A- \left(C +D\right)$$

To estimate whether the number of smokers introduced into the UK population because of flavoured e-liquid availability could outweigh the number of smokers and ex-smokers who might vape instead of smoke because of flavoured e-liquid availability, the following calculation is made:

$$B- \left(C +D\right)$$

Positive values would provide some support for a total ban on flavoured e-liquids, and negative values would provide some evidence that the detrimental consequences of a total ban would outweigh the potential benefits.

### Data sources

In November 2024, we entered data from Action on Smoking and Health (ASH) surveys [2, 22] (collected between February and April 2024) and the Smoking Toolkit Study (STS; collected between June and October 2023) [23]. ASH data are collected via yearly online surveys in Spring by YouGov. STS surveys are conducted by Ipsos via monthly telephone interviews. Alongside the survey data, we input publicly available figures from the Office of National Statistics (ONS) [24, 25] and privately owned real-time statistics website Worldometer (estimates taken on 13 November 2024) [26]. The exact figures, weights, sources, survey questions and code used to determine the data input can be found on GitHub (https://github.com/jkhouj/PolicyDecisionAid).

### Survey populations

ASH and STS conduct surveys in Great Britain (which includes England, Scotland and Wales) and England, respectively. We combined estimates from ASH and STS using weighted proportions. These were then scaled to UK population totals because no Northern Ireland data were available. Approximately half of the respondents across the surveys were male, and about a third were from more disadvantaged social grades according to the National Readership Survey.

### Youth and adult definitions

ASH collect data from respondents aged 11–18 years in a youth survey and respondents aged 18+ years in an adult survey, with each survey using distinct questions. In our model, those who completed the youth survey were classified as “youth”, and those who completed the adult survey were classified as “adults”. Although the survey age ranges overlap, each respondent contributed only to either the youth or adult estimates, ensuring no double counting.

STS surveys collect data from respondents aged 16+. During consultation, policymakers and research experts advised that we should classify individuals up to age 20 years as youth, given that vaping initiation and potential gateway effects could extend beyond age 18 years. We therefore stratified the STS data by age into “youth” (16–20 years) and “adult” (21+).

For the purposes of the model, the proportions calculated on the basis of ASH and STS youth data were scaled to the number of 11–20 year olds in the UK, and the ASH and STS adult data were scaled to the number of adults aged 18+ with a smoking history in the UK.

### Survey weighting

ASH data were weighted to be representative of children (aged 11–18 years) and adults 18+ in Great Britain [2, 22] by multiplying percentage estimates by the survey weights provided. STS data were weighted to be representative of people aged 16 years and older in England using the rim (marginal) weighting technique, as previously described [23]. The percentage estimates were multiplied by the survey weights to be representative of the population in terms of gender, working status, prevalence of children in the household, age, social grade and region.

### Survey missingness

All respondents had complete smoking history data in the ASH and STS surveys. Some survey questions were only asked amongst subsets of respondents who met prespecified criteria. Because the missingness was partly due to complex survey logic rather than random processes, imputation could have introduced bias; therefore, we retained complete-case data to estimate proportions.

## Results

**Fig. 1:**
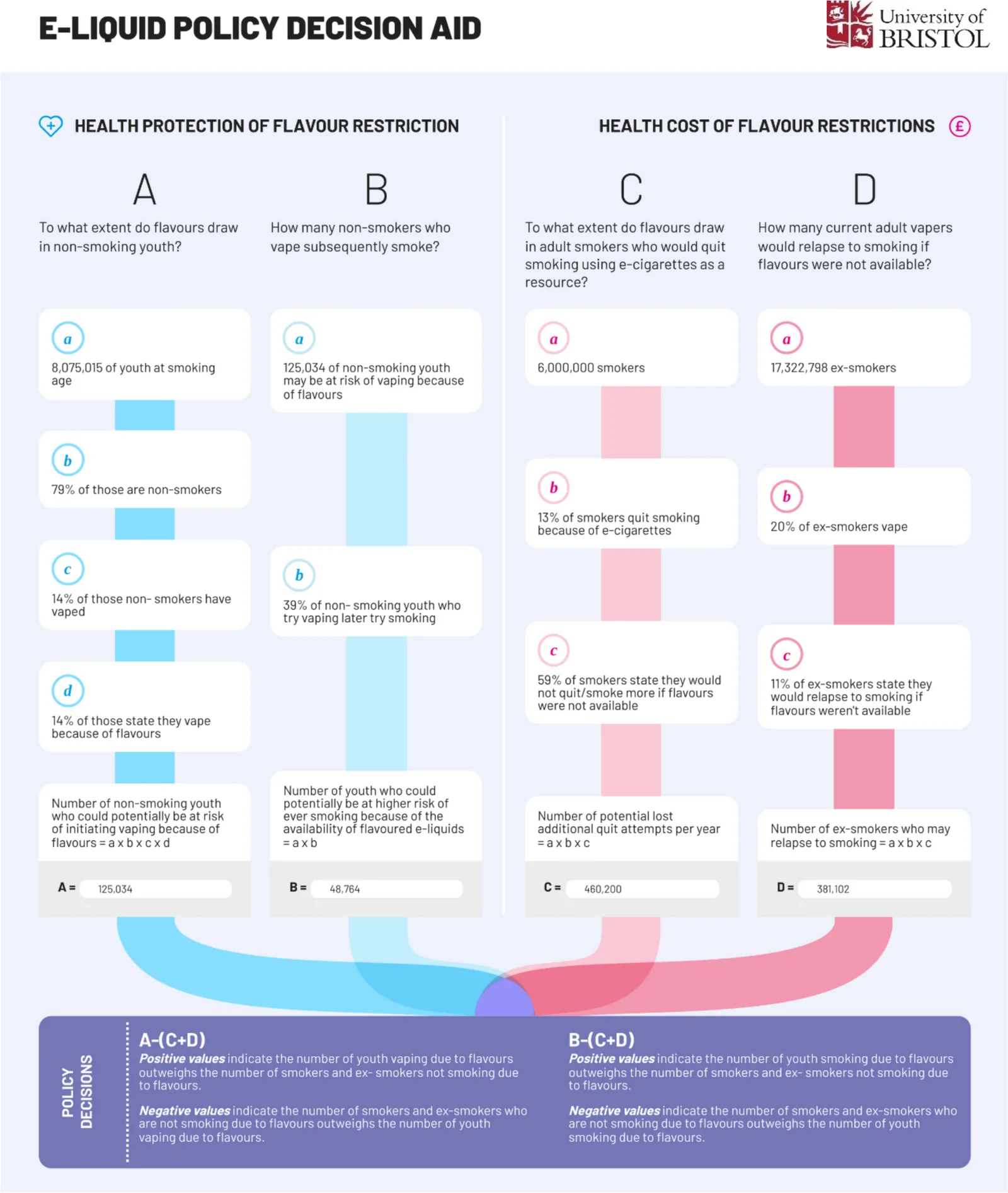
Policy decision aid report to support policymaking on flavour restriction to reduce youth vaping. This report displays the November 2024 output

Using the available evidence on 13 November 2024, we estimated that there were 8 075 015 young people aged 11–20 years in the United Kingdom by scaling the proportion of 11–20 year olds in the 2021 census (11.654%) to the current size of the UK population (*N* = 69 291 190). Combining weighted data from the ASH 2024 youth survey (*N* = 2872 respondents aged 11–18 years) and STS 2023 surveys (*N* = 541 respondents aged 16–20 years), we estimated that 79% had never smoked, 14% of those had never smoked but had ever vaped and 14% of those had stated they vape because they “like the flavours”. Using the algorithm (A), we estimated that 125 034 non-smoking youth experiment with e-cigarettes as a result of flavoured e-liquid availability (Fig. 1). Under the assumptions of the decision aid, we also estimated that 48 764 non-smoking youth subsequently smoke as a result of flavoured e-liquid availability by multiplying the A estimation by the weighted proportion of those who smoked after vaping versus those who had vaped but never smoked (39%) using algorithm B (Fig. 1). The ONS estimated that 6 million adults in the United Kingdom were current smokers in 2023 and that 25% were ex-smokers; scaling this to the UK population size in 2024, we estimated there were 17 322 798 ex-smokers. Combining weighted data from the ASH 2024 adult survey (*N* = 13 266 aged 18–94 years) and STS 2023 surveys (*N* = 11 423 respondents aged 21–99 years), we estimated that 13% of adult smokers had quit in the past year using e-cigarettes, and 59% of current adult dual users and those who had quit within the last year using an e-cigarette stated they would “smoke more tobacco” or “go back to smoking tobacco” if flavours became unavailable. We also estimated that 20% of ex-smokers currently vaped, and 11% of those stated they would “smoke more tobacco” or “go back to smoking tobacco” if flavours became unavailable. Using the algorithm (C and D), we estimated that 841 302 smokers and ex-smokers do not smoke due to flavoured e-liquid availability (Fig. 1). This output suggests that restricting flavoured e-liquids in the United Kingdom could have a detrimental overall impact on public health when considering both youth vaping and smoking uptake due to flavoured vape availability. The full report (Additional File 1) was shared with the Office of Health Improvements and Disparities.

## Discussion

Herein we have illustrated the potential of a policy decision aid to assist policymakers in deciding whether to ban flavours in vapes. The report in November 2024 (and all prior reports) indicates that banning flavours in e-cigarettes would detrimentally impact smoking cessation and abstinence rates in the United Kingdom more than it would prevent youth uptake of vaping and smoking.

The findings of this and earlier outputs of this decision aid (with the same broad conclusions) have been discussed in the UK Parliament, cited in responses to calls for evidence [27] and cited in the UK government Tobacco and Vapes Bill Impact Assessment conducted by the Department of Health and Social Care (https://publications.parliament.uk/pa/bills/cbill/59-01/0121/impactassessment.pdf). The impact assessment concluded (in line with the evidence we provided) that 87% of adults who vape could be impacted. The assessment also highlighted that the outputs echo the findings of Friedman and colleagues [15] whereby reductions in the sale of vapes following flavour restrictions coincided with increases in tobacco sales. Our previous work using this model to estimate the impact on the general United States population also indicated that there would be an increase in smoking rates [28].

The findings are also in line with our prior qualitative research, where participants described their intentions to smoke more in the hypothetical scenario where flavours are banned in the United Kingdom [10]. However, a review of anticipated and actual responses to banning flavours in e-cigarettes has shown mixed findings regarding the effectiveness and unintended consequences of a ban [29]. Although some of the evidence in the review contradicts the output from the policy decision aid, the review conclusions relied on evidence from the United States which may not apply to the United Kingdom given the different social and regulatory environments (e.g. flavoured tobacco products were banned at the same time as flavoured e-cigarettes whereas flavoured cigarettes, including menthol, have been banned since 2020 in the United Kingdom).

The use of the decision aid output in government discussions, reports and impact assessments demonstrates that it is accessible for policymakers. The output is also relatively quick to produce when new data become available, meaning we have been able to supply policymakers with eight updates in 3 years. However, data availability has been scarce and thus far required additional funding for data collection, as national surveys do not routinely include questions about flavours and e-cigarette flavour restrictions.

The output is also intended to guide policy decisions in the context of other available evidence, and is not to be used as the sole determining factor when making policy decisions due to the simplicity of the model and potential bias due to sampling biases in the data collected. If no relevant evidence is available, the decision aid output could be used to guide governmental support for future research by highlighting areas for research. For example, if the tool showed a beneficial impact when including menthol and tobacco flavours versus excluding them from the ban, the government could support further research in this area to improve the evidence base for this policy decision.

The simplistic model is necessary to provide the timely estimates that policymakers require [8], but consequently, the output cannot account for many nuances in behaviour. For example, the decision aid does not consider the impact of the role of e-cigarettes in displacing cigarette smoking amongst young people or of second-hand smoke exposure. Additionally, the model assumes that e-cigarettes act as a gateway to smoking, as this is a concern for policymakers. However, despite the consistent association between vaping and later smoking amongst young people and adults, it is unlikely that the gateway hypothesis provides a complete explanation for the association; there is substantial evidence that the relationship is partly explained by a shared liability to use either product, perhaps due to an underlying genetic propensity for risk-taking [4, 30, 31]. This decision aid provides a worst-case scenario for policymakers given their concerns, but may overestimate the harm to youth. Furthermore, we focussed on cigarette use specifically, however, any vaping regulations could impact other nicotine and tobacco use [10, 32]. The use of other combustible products in England is rare (< 2% prevalence in 2023), but there has been a substantial increase in recent years, particularly amongst young people [33]. Future amendments to the decision aid may need to address this.

The data used are also restricted to respondents from Great Britain, and although we have scaled this to the United Kingdom, there may be differential impacts in Northern Ireland that are not accounted for. Furthermore, there is some overlap in the age of our adult and youth groups; ASH collected youth data on 11–18 year olds and adult data on 18+ years, whereas we stratified the STS data for youth on 16–20 year olds and for adults on 21+ given that initiation of e-cigarettes and cigarettes is occurring later in life than in previous generations [34, 35] and policymakers were concerned about young adult behaviour. The youth responses were then scaled to the number of 11–20 year olds in the United Kingdom, and the adult responses were scaled to the estimated number of current and ex-smokers who are 18 years or older in the United Kingdom. Therefore, the estimates could be an overestimation whereby young adults aged 18–20 years who vaped before they smoked and have transitioned between smoking and vaping multiple times may be included in both the estimation of B and either C or D.

Finally, the survey questions that underly the data used in this tool estimate anticipated behaviour in the event of a flavour ban and could therefore underestimate individuals’ ability to refrain from smoking. However, until a ban is introduced, it will not be possible to observe actual behaviour in the United Kingdom’s unique social and policy context.

As highlighted by these limitations, effective and timely policy communication requires researchers to provide less complex, precise and nuanced results in favour of providing results that can be easily interpreted by policymakers. This framework can be adapted to explore the potential impact on different subpopulations and the impact of flavour restrictions in other localities. Additionally, the tool can be adapted to answer other policy questions, where there is a tradeoff between policies protecting some individuals but potentially harming others. However, such adaptations need to identify the framework limitations in the context of the policy question, as they will likely differ from the limitations identified here.

## Conclusions

When provided with simple, easy-to-digest information that is relevant to the current policy environment, policymakers can synthesize evidence from public health researchers to guide policy development.

## Supplementary Information

Supplementary Material 1.

## Data Availability

The data supporting the conclusions of this article are available in the GitHub repository, https://github.com/jkhouj/PolicyDecisionAid. For access to the original STS data, please use these forms: https://smokinginengland.info/resources/data-request-agreement-form; https://smokinginengland.info/resources/data-request-coi-form. For access to ASH YouGov data, please contact [enquiries@ash.org.uk] (mailto:enquiries@ash.org.uk).

## Abbreviations

ASH: Action on Smoking and Health
ONS: Office of National Statistics
STS: Smoking Toolkit Study
UK: United Kingdom
